# Image-guided injections for facet joint pain: evidence-based Delphi conjoined consensus paper from the European Society of Musculoskeletal Radiology and European Society of Neuroradiology

**DOI:** 10.1007/s00330-025-11651-9

**Published:** 2025-05-08

**Authors:** Luca Maria Sconfienza, Danoob Dalili, Miraude Adriaensen, Domenico Albano, Georgina Allen, Maria Pilar Aparisi Gomez, Giacomo Aringhieri, Francesco Arrigoni, Alberto Bazzocchi, Miguel Oliveira Castro, Roberto Luigi Cazzato, Miriam De Dea, Aldo Eros De Vivo, Elena Drakonaki, Fernando Facal de Castro, Dimitrios Filippiadis, Jan Fritz, Inês Gil, Salvatore Gitto, Hannes Gruber, Harun Gupta, Amanda Isaac, Andrea S. Klauser, Thomas Le Corroller, Alexander Loizides, Salvatore Marsico, Giovanni Mauri, Eugene McNally, Kalliopi Melaki, Carmelo Messina, Rebeca Mirón Mombiela, Cyprian Olchowy, Davide Orlandi, Ricardo Moutinho, Riccardo Picasso, Mahesh Prakash, Nicolas Theumann, Violeta Vasilevska Nikodinovska, Evangelia E. Vassalou, Jelena Vucetic, David Wilson, Federico Zaottini, Marcello Zappia, Chiara Zini, Žiga Snoj

**Affiliations:** 1 IRCCS Istituto Ortopedico Galeazzi, Milano, Italy; 2 Dipartimento di Scienze Biomediche per la Salute, Università degli Studi di Milano, Milano, Italy; 3 Epsom and St. Helier Hospitals, Surrey, London, United Kingdom; 4 Department of Medical Imaging, Zuyderland Medical Center, Sittard-Geleen, Heerlen, Brunssum, Kerkrade, The Netherlands; 5 Dipartimento di Scienze Biomediche, Chirurgiche e Odontoiatriche, Università degli Studi di Milano, Milano, Italy; 6 St Luke’s Radiology Oxford UK Ltd, Oxford, UK; 7 University of Oxford, Oxford, UK; 8 Department of Radiology, Te Toka Tumai Auckland (Auckland District Health Board), Auckland, New Zealand; 9 Department of Anatomy and Medical Imaging, Faculty of Medical and Health Sciences, Waipapa Taumata Rau, University of Auckland, Auckland, New Zealand; 10 Department of Radiology, IMSKE, Valencia, Spain; 11 Academic Radiology, Department of Translational Research, University of Pisa, Pisa, Italy; 12 Department of Biotechnological and Applied Clinical Sciences, University of L’Aquila, L’Aquila, Italy; 13 IRCCS Istituto Ortopedico Rizzoli, Bologna, Italy; 14 Department of Radiology, ULS Algarve, Portimão, Portugal; 15 University Hospital of Strasbourg, Strasbourg, France; 16 Studio MSK, Belluno, Italy; 17 IOM Istituto Oncologico del Mediterraneo, Catania, Italy; 18 University of Crete Medical School, Giofirakia, Greece; 19 Iberorad 1895 S.L., Barcelona, Spain; 20 2nd Department of Radiology, University General Hospital “ATTIKON” Medical School, National and Kapodistrian University of Athens, Athens, Greece; 21 Department of Radiology, NYU Grossman School of Medicine, New York, USA; 22 Diagnostic and Interventional Radiology, Eberhard Karls University Tuebingen, University Hospital Tuebingen, Tübingen, Germany; 23 Department of Radiology, ULS Algarve, Faro, Portugal; 24 Medical University Innsbruck - Tirol Kliniken, Innsbruck, Austria; 25 Leeds Teaching Hospitals, Leeds, UK; 26 King’s College London, London, UK; 27 Department of Radiology, Medical University Innsbruck, Innsbruck, Austria; 28 Aix-Marseille Université, Marseille, France; 29 Hospital del Mar, Barcelona, Spain; 30 Oxford MSK Radiology, Oxford, UK; 31 KMG Klinikum Luckenwalde-Akademisches Lehrkrankenhaus des Universitätsklinikums der Martin-Luther-Universität Halle-Wittenberg, Luckenwalde, Germany; 32 U.O.C. Radiodiagnostica, ASST Centro Specialistico Ortopedico Traumatologico Gaetano Pini-CTO, Milano, Italy; 33 Radiology Department, Herlev og Gentofte Hospital, Herlev, Denmark; 34 Collegium Medicum, Jan Dlugosz University in Czestochowa, Czestochowa, Poland; 35 Department of Radiology, Ospedale Evangelico Internazionale, Genova, Italy; 36 Hospital da Luz, Musculoskeletal Imaging Unit, Lisbon, Portugal; 37 Hospital de Loulé, Loulé, Portugal; 38 IRCCS Ospedale Policlinico San Martino, Genova, Italy; 39 Department of Radiodiagnosis, PGIMER, Chandigarh, India; 40 HIRSLANDEN LAUSANNE SA, Lausanne, Switzerland; 41 Ss.Cyril and Methodius University, Skopje, Macedonia; 42 University Surgical Clinic “St.Naum Ohridski”, Skopje, Macedonia; 43 University Hospital of Heraklion, Heraklion, Greece; 44 Radiology Department, Hospital ICOT Ciudad de Telde, Las Palmas, Spain; 45 University of Molise, Campobasso, Italy; 46 Department of Radiology, Azienda USL Toscana Centro, Firenze, Italy; 47 Institute of Radiology, University Medical Centre Ljubljana, Ljubljana, Slovenia

**Keywords:** Spine, Facet joints, Interventional radiology, Injection, Delphi process

## Abstract

**Objectives:**

To perform a Delphi-based consensus on published evidence on image-guided injections for facet joint pain (FJP) and provide clinical indications.

**Methods:**

We report the results of an evidence-based Delphi consensus of 38 experts from the European Society of Musculoskeletal Radiology and the European Society of Neuroradiology, who reviewed the published literature for evidence on image-guided injections for FJP. Experts drafted a list of statements and graded them according to the Oxford Centre for evidence-based medicine levels of evidence. Consensus was considered strong when ≥ 95% of experts agreed with the statement or broad when > 80% but < 95% agreed. The results of the consensus were used to write the paper.

**Results:**

Twenty statements on image-guided FJP treatment have been drafted. Eighteen statements received strong consensus, while two received broad consensus. Three statements reached the highest level of evidence, all of them regarding the lumbar spine. All radiological methods are used for image-guided injections for FJP, and regardless of the radiological method used, all show good safety and efficacy. Facet joint injections and medial branch blocks are used in all spinal regions to treat FJP, and both show similar clinical outcomes. Advanced technological solutions have been studied in the field of lumbar FJP; however, the level of evidence for these is low.

**Conclusion:**

Despite promising results reported by published papers on image-guided injections for FJP, there is still a lack of evidence on injection efficacy, appropriateness of imaging methods, and optimal medication.

**Key Points:**

***Question*** *Image-guided injections to treat facet joint pain (FJP) are performed throughout the spine; however, the highest level of evidence exists for the lumbar spine.*

***Findings*** *Regardless of the imaging method used, image-guided injections for facet joint pain treatment are safe, with only minor adverse events in rare cases.*

***Clinical relevance*** *All imaging methods are used for injection guidance to treat FJP, each with advantages and disadvantages. These statements on image-guided injections for FJP provide a concise and up to date overview on the topic, serving as a list of clinical indications.*

**Graphical Abstract:**

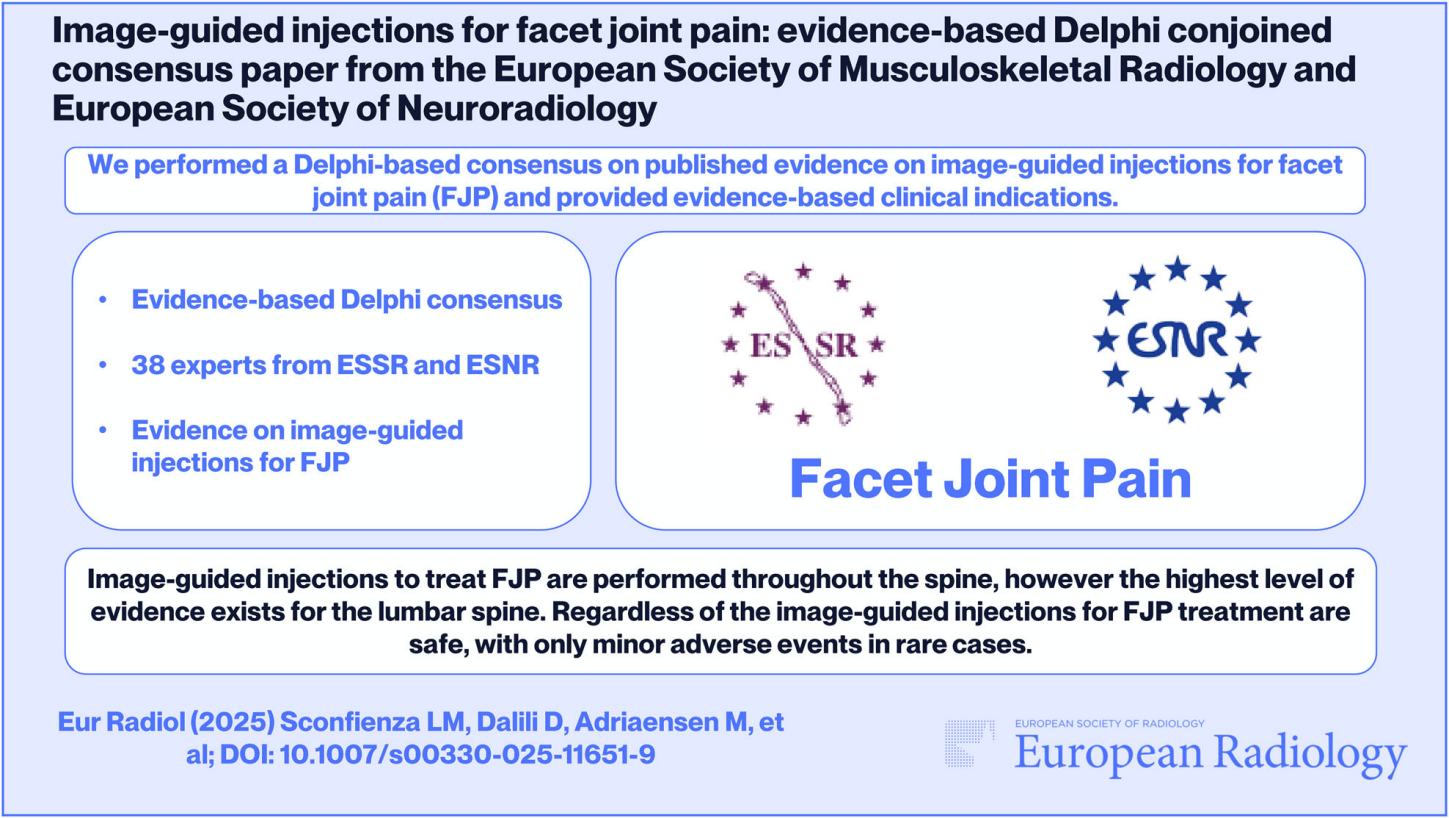

## Introduction

Chronic back pain is very common, and it is a frequent cause for patients to seek medical advice [1]. It is also a major cause of disability and work absence, thus representing a significant health and societal issue [2]. 

In the spine, facet joints are one of the most important, albeit neglected, pain generators [3]. The lumbar tract is the most frequently involved, with a lifetime pain prevalence of approximately 40% [4].

Treatment of facet joint-originated pain is not standardized. Initial conservative approaches involve the use of oral anti-inflammatory/myorelaxant drugs, physiotherapy, weight loss, or their combination, mostly based on the physician’s personal experience rather than guidelines [3]. Local therapies can be used to target the intervention and reduce systemic side effects of oral medications. By reducing local pain, injections may help improve rehabilitation. Image guidance targeting and reducing complications [5].

The European Society of Musculoskeletal Radiology (ESSR) and the European Society of Neuroradiology (ESNR) experts reviewed the current literature on image-guided injections to treat facet joint pain (FJP) and drafted a list of evidence-based statements.

## Materials and methods

Ethical Committee approval was not required as no patients were involved. This paper is part of a larger project established by the ESSR Intervention Subcommittee and the ESNR Spine Committee to assess the published evidence on image-guided interventional procedures around the spine and to produce a list of clinical indications [6–12]. An expert panel, selected from experienced members of these subcommittees, evaluated the existing literature, using the same methods as reported for other regions of the body in the ESSR consensus papers [6–12]. The Delphi method consisted of rounds of literature evaluations by a panel of experts to create a list of agreed indications for each topic [13]. The AGREE II tool was used to ensure the quality of the Delphi method and the resulting guidelines [14]. The complete process is detailed in the Supplementary Material.

## Results

### 1. US-guided injections offer better clinical outcomes than blind injections in patients with lumbar FJP

Level of Evidence (LoE): 2

A randomized controlled trial studied patients with lumbar FJP, showing better clinical outcomes in a US-guided injection group versus a blind local injection group up to 6 weeks [15]. The long-term outcome remains unknown.

Agree = 38/38; 100%

Disagree = 0/38; 0%

### 2. Medial branch block (MBB) with local anesthetic and corticosteroid offers similar clinical outcomes as MBB with local anesthetics only

LoE: 2

Randomized controlled trials on cervical, thoracic, and lumbar spine showed comparable outcomes after fluoroscopy-guided facet joint injection (FJI) of local anesthetic with corticosteroid or local anesthetic only [16–18]. Studies showed good pain management by repetitive injections up to two years [17, 18]. Participants received up to 9 injections in a 2-year timeframe [17, 18].

Agree = 36/38; 95%

Disagree = 2/38; 5%

### 3. US-guided FJI of a mixture of methylene blue and lidocaine is safe and effective in patients with lumbar FJP

LoE: 2

A prospective randomized controlled trial showed that US-guided intra-articular injection of methylene blue and lidocaine is safe and effective in patients with lumbar FJP [19]. The authors compared the outcomes of two groups, one receiving methylene blue and lidocaine injection and another receiving corticosteroid and lidocaine injection. At 1 month, there was no difference between the groups, but methylene blue and lidocaine performed significantly better at 3 and 6 months [19]. Adverse events were similar in both groups, and there was no abnormal liver/kidney function up to 6 months [19].

Agree = 36/38; 95%

Disagree = 2/38; 5%

### 4. US-guided cervical MBB is quicker and requires fewer needle passes than fluoroscopic guidance

LoE: 2

A randomized control trial showed shorter procedure times and fewer needle passes for US-guided MBB compared to fluoroscopy-guided MBB at the C7 level [20]. A retrospective study on MBB on different cervical levels [21] published supporting evidence. A further advantage of US-guided over fluoroscopy-guided MBB is the lack of radiation exposure [21].

Agree = 35/38; 92%

Disagree = 3/38; 8%

### 5. There is no clinical outcome difference between US-guided, fluoroscopy-guided, and CT-guided cervical FJI and MBB

LoE: 2

A retrospective study compared US-guided versus fluoroscopy-guided cervical MBB in patients with cervical FJP [21]. C2–C7 levels were injected, and no differences in functional improvement and analgesic effect for up to 6 months were found for the different radiological methods [21]. A randomized control trial also showed no pain relief differences between US-guided versus fluoroscopy-guided MBB at C7 20 min after injection [20]. A prospective randomized trial showed similar clinical outcomes up to 1 month after US-guided FJI and CT-guided FJI for levels from C2/C3 to C7/Th1 [22].

Agree = 36/38; 95%

Disagree = 2/38; 5%

### 6. US guidance offers accuracy similar to fluoroscopy for cervical MBB

LoE: 3

A study performed US-guided cervical MBB using a coronal view of the articular pillar with an out-of-plane approach and reported a success rate of 87%. Another study used a transverse view of the articular pillar with an in-plane, posterolateral approach and reported a success rate approaching 95%, stating that the latter approach is safer based on anatomical considerations [23]. In both studies, the accuracy rate decreased for lower segments, with a significant difference only found at C7 by Siegenthaler et al [23, 24]. They concluded that US guidance offers high accuracy of cervical MBB except for C7 level [24]. However, another study showed a success rate of 96% for US-guided MBB at C7 level [20].

Agree = 35/38; 92%

Disagree = 3/38; 8%

### 7. Lateral US-guided approach is more accurate than the posterior approach for cervical FJI

LoE: 3

Two studies verified the lateral US-guided approach using CT or fluoroscopy and reported 92% and 100% accuracy rates, respectively [25, 26]. A cadaver study evaluated a posterior US-guided approach and reported a 78% accuracy rate [26]. The authors postulated that the lower accuracy of the posterior approach might be related to deeper joint location compared to the lateral approach, making it more difficult to identify [25].

Agree = 38/38; 100%

Disagree = 0/38; 0%

### 8. There is no difference in outcome between thoracic FJI and MBB under fluoroscopy guidance

LoE: 2

A randomized control trial compared the clinical outcome of thoracic FJI versus MBB under fluoroscopy guidance [27], showing significant improvement up to 6 months after both procedures, with no clinical differences [27]. Other studies also confirmed the efficacy of MBB under fluoroscopy guidance up to 3 years follow-up [17, 28, 29].

Agree = 38/38; 100%

Disagree = 0/38; 0%

### 9. There is no time duration difference between US-guided, fluoroscopy-guided, and CT-guided lumbar FJI and MBB

LoE: 1

A systematic review and meta-analysis reviewed studies comparing US-guided to fluoroscopy-guided and CT-guided MBB and FJI procedure time, and differences were statistically insignificant [30, 31]. However, the definition and measurement of procedure time varied across studies [31]. A meta-analysis reported a moderate risk of bias from selection bias, imprecision because of relatively small samples, and high inconsistency from lack of a priori statistics [31].

Agree = 38/38; 100%

Disagree = 0/38; 0%

### 10. There is no difference in outcome between lumbar FJI and MBB under fluoroscopy guidance

LoE: 2

A randomized controlled study compared clinical outcomes between lumbar FJI and MBB under fluoroscopy guidance for up to 6 months [32]. The results showed comparable outcomes between groups at all time points [32]. Similar findings were described in a randomized controlled study with a three-month follow-up [33].

Agree = 38/38; 100%

Disagree = 0/38; 0%

### 11. US-guided lumbar FJI is as safe and effective as fluoroscopic- and CT-guided FJI

LoE: 1

A meta-analysis showed no significant differences in pain and functional improvement among US-guided and fluoroscopy-guided or CT-guided injections [34]. A randomized controlled trial showed no difference between US-guided and fluoroscopy-guided injections in pain relief and daily life activities for up to 3 months [35]. Two further studies confirmed these findings, and both procedures showed analgesic effects up to 6 months with no patient outcome differences between different guidance methods [36, 37].

Agree = 37/38; 97%

Disagree = 1/38; 3%

### 12. In lumbar FJI and MBB, needle positioning under US guidance is less accurate than using fluoroscopy or CT

LoE: 1

A meta-analysis showed that the risk of incorrect needle placement using US guidance for lumbar FJI and MBB is high when needle placement is verified with CT or fluoroscopy [31]. The meta-analysis did not consider studies in which the US was associated with other radiological methods using fusion imaging. It also reported a moderate risk of bias from selection bias, imprecision because of relatively small samples, and high inconsistency from lack of a priori statistics [31]. When a study with a cohort of obese patients was removed from analysis, the difference was less pronounced but still significant [38]. Besides obesity, spondylolisthesis also hinders the accuracy of US-guided MBB [38, 39].

Agree = 36/38; 95%

Disagree = 2/38; 5%

### 13. There is no difference in the 1-month outcome between fluoroscopy-guided and CT-guided lumbar FJI

LoE: 3

A study compared the outcome of fluoroscopy versus CT guidance for lumbar FJI in 599 patients [40]. There were no outcome differences up to 1-month follow-up [40].

Agree = 38/38; 100%

Disagree = 0/38; 0%

### 14. Radiation exposure in fluoroscopy-guided lumbar FJI is lower for patients and higher for physicians when compared with CT guidance

LoE: 3

A study reported 3.3 times lower patient radiation dose exposure for fluoroscopy-guided FJI than CT guidance [40]. Conversely, radiation exposure to interventional physicians’ bodies and wrists was higher for fluoroscopy-guided FJI than CT guidance [40].

Agree = 38/38; 100%

Disagree = 0/38; 0%

### 15. Physician experience, physician control of fluoroscopy, and anteroposterior approach reduce radiation exposure in fluoroscopy-guided lumbar MBB

LoE: 3

A study compared two approaches of fluoroscopy-guided lumbar facet MBB [41]. It showed that the anteroposterior approach, compared to the oblique approach, reduces radiation exposure and fluoroscopy time in fluoroscopy-guided lumbar MBB. Also, the physician’s experience positively impacted these outcomes [41]. Another study evaluated the difference of fluoroscopy time and radiation dose depending on the medical staff operating the system in spine interventions [42]. They showed that fluoroscopy time and radiation dose were significantly decreased when the physician directly controlled the fluoroscopy unit versus the radiographer controlling the system [42].

Agree = 38/38; 100%

Disagree = 0/38; 0%

### 16. Adverse events during image-guided FJI are uncommon and minor

LoE: 3

Two large studies investigated adverse events in fluoroscopy-guided FJI [43, 44], including 43,010 and 11,980 FJIs [43, 44]. Manchikanti et al reported intravascular injection in 11.4% of cases overall (20% cervical spine, 4% lumbar spine, 6% thoracic spine), significantly higher in the cervical region per encounter/episode [43]; local hematoma was seen in 1.2% of patients; bruising, soreness, nerve root irritation, and all other complications were observed in < 1% of cases [43]. Kim et al reported a 0.83% overall incidence rate/procedure. The most frequent adverse events requiring hospitalization/emergency room visits were post-procedural pain exacerbation in 0.52% of patients, followed by spinal infection in 0.07% of patients [44]. A review reported only minor adverse events such as vasovagal reactions, transient headaches, superficial hematomas, and superficial infections [31]. Only sparse case reports highlighted severe and major adverse events after lumbar FJI [45–47].

Agree = 38/38; 100%

Disagree = 0/38; 0%

### 17. MRI-guided FJI are safe, feasible, and effective to treat lumbar FJP

LoE: 3

Feasibility and safety of MRI-guided FJI have been proven on different MRI systems of 0.2–1.5 T field strengths [48–50]. A study reported pain relief up to 12 months after MRI-guided FJI [50]. However, wide clinical adoption is still limited.

Agree = 38/38; 100%

Disagree = 0/38; 0%

### 18. MRI-US and CT-US fusion-guided injections are safe and effective to treat lumbar FJP

LoE: 3

For lumbar FJP treatment, the safety and efficacy of MRI-US [51] and CT-US [52] fusion-guided injections have been proven. Both papers reported no clinical difference between the fusion and non-fusion groups up to 2 or 6 months, respectively [52]. No major complications were noted [51, 52]. Several systems for fusion and guidance have been validated with targeting accuracy as low as 0.6 mm, but clinical adoption is still limited [53–55].

Agree = 37/38; 97%

Disagree = 1/38; 3%

### 19. Augmented reality-guided lumbar FJI shows promising results, especially as a learning tool, but its clinical value has not been demonstrated

LoE:4

Augmented reality-guided FJI has been validated with both MR and CT guidance [56] These studies have proven accurate needle placement in the experimental setting on phantoms [56, 57]. However, there are studies on the clinical application of augmented reality for FJI [56, 57]. This application may assist medical trainees in acquiring technical competence [58].

Agree = 38/38; 100%

Disagree = 0/38; 0%

### 20. Robotic-guided lumbar FJI shows promising results, but clinical value is not demonstrated

LoE:4

The feasibility of robotic US-based navigation and needle guidance for lumbar FJI has been reported [59]. Another study showed that robotic-assisted FJI allows accurate positioning with minor needle adjustments compared to the freehand approach [60]. Although robotic-assisted injections took longer than the freehand approach, they were still fast. Authors believe that the advantage of no need for needle repositioning outweighs the impact of slightly longer procedure time [60]. Both studies used phantoms and validated needle insertion accuracy with either cone-beam CT or CT [59, 60].

Agree = 38/38; 100%

Disagree = 0/38; 0%

## Discussion

After a Delphi-based consensus process, the ESSR/ESNR expert group drafted twenty evidence-based statements regarding image-guided interventional procedures to treat FJP. Only three statements reached the highest level of evidence. Eighteen statements received a strong consensus, while two had a broad consensus. These data reflect the existing controversies on the radiological method of choice for image guidance, as both statements with broad consensus were on cervical MBB, comparing the accuracy and timeframe of US versus fluoroscopy guidance. All radiological methods are used for the guidance of FJI, with fluoroscopy being the most common method [5]. Conversely, MR is the least used radiological method; thus, data on MR-guided procedures are scarce due to higher costs of systems and dedicated tools and limited availability [61].

US-guided facet joint injection has demonstrated superior clinical outcomes compared to blind injections (statement #1) [15]. This is similar to other body regions [7, 8]. This is important not just for the clinical outcome but also for the correct diagnosis of FJP, which is still debated [5].

Highest level of evidence was reached for statements on lumbar FJI. Despite the fact that US-guided lumbar FJI and MBB are less accurate than fluoroscopy- or CT-guided injections, there is no safety and effectiveness difference among radiological methods (statements #11 and #12) [34–38]. This is likely linked to the results of studies reporting no clinical difference between FJI and MBB (statement #10) [32, 33]. Also, FJI and MBB are often dealt with as equivalent in literature, lacking clear differentiation and reporting [30]. Individual patient characteristics, such as high BMI and advanced degenerative disease, have a negative effect on the accuracy of US-guided injections [31, 38]. However, in selected patients, US may outperform other radiological methods, especially when patient positioning on a CT or C-arm table is difficult, as in patients with neuromuscular disorders that have severe kyphoscoliosis [62]. Ionizing radiation is a known disadvantage of CT and fluoroscopy guidance; however, fluoroscopy allows detection of inadvertent vascular injection [63, 64]. Fluoroscopy and CT guidance offer similar clinical outcome for lumbar FJI (statements #13 and #14) [40], but minimal adaptations may reduce radiation exposure during fluoroscopy usage (statement #15) [41]. Physician preference and professional background also play a role in imaging method choice, as the US may have a longer learning curve. However, non-radiating radiological methods should always be preferred [65], and radiologists should master them to improve patient care [66].

A statement on equivalent procedure time among US-, fluoroscopy-, and CT-guided lumbar FJI and MBB reached the highest level of evidence (statement #9) [30, 31]. However, this is not the case for US-guided cervical injections (statement #4) [20]. This can be explained with shallower target depth and better capacity to visualize the anatomy in the cervical versus lumbar spine. In cervical spine, these considerations are important to choose a more accurate approach (#7) [25, 26]. Cervical US-guided injections show good accuracy and similar clinical outcomes compared to fluoroscopy or CT guidance (statements #5 and #6) [20–24]. The authors emphasized important US advantages, such as real-time in-plane needle visualization [22, 67].

Thoracic spine procedures have very little evidence, reflecting the lower incidence of dorsal pain compared to other segments [68]. However, thoracic procedures under fluoroscopy guidance offer good outcomes (statement #8) [27–29]. For US-guided FJI, only technical papers are available [69]. Authors underline the risk of segment misidentification, especially if the C7 spinous process served as an initial landmark [70].

Technological advancements in FJP treatment have been reported, but evidence is low (statements #18, #19, and #20) [51–60]. Fusion technologies have been proven safe and effective for lumbar FJI [51–55], while augmented reality-guided [56–58] and robotic-guided FJI [59, 60] showed promising results but are limited to experimental studies. Overall, all of them require additional hardware/software and time for setup.

Overall, studies reported no clinical differences for MBB between using local anesthetic with steroids or local anesthetic only (statement #2) [16–18]. Notably, one study used methylene blue and lidocaine for lumbar FJI, stating its superiority compared to steroids. However, the association of methylene blue with lidocaine prevents a specific assessment of the efficacy of this novel injectate (statement #3) [19].

Some limitations should be considered. First, statements are not a detailed meta-analysis, the study design is an expert opinion that led us to draft a consensus document. Delphi-based method was used for review of the existing literature, for experts’ consensus gathering and to establish future directions to increase evidence on this topic. Also, no statistical analysis was performed. Last, other types of treatments than injections are available to treat FJP, which were beyond this paper’s scope.

In conclusion, the ESSR/ESNR expert panel produced twenty evidence-based statements on image-guided injections for FJP and MBB. Three statements achieved the highest level of evidence; all of these were associated with image-guided injections for lumbar FJP. Overall, the level of evidence remains limited; thus, further larger prospective randomized trials are warranted.

## Supplementary information

ELECTRONIC SUPPLEMENTARY MATERIAL

## Abbreviations

ESNR: European Society of Neuroradiology
ESSR: European Society of Musculoskeletal Radiology
FJI: Facet joint injection
FJP: Facet joint pain
Loe: Level of evidence
MBB: Medial branch block
