# Supplementary material for: Image-guided injections for facet joint pain: evidence-based Delphi conjoined consensus paper from the European Society of Musculoskeletal Radiology and European Society of Neuroradiology
Source: Eur Radiol. 2025 May 8;35(11):6958–66. doi: 10.1007/s00330-025-11651-9 (PMC12559049; doi:10.1007/s00330-025-11651-9)
Supplement: Supplementary file 1 — ELECTRONIC SUPPLEMENTARY MATERIAL [file 330_2025_11651_MOESM1_ESM.pdf]

Image-guided injections for facet joint pain: evidence-based Delphi conjoined consensus paper from the European Society of Musculoskeletal Radiology and European Society of Neuroradiology
ELECTRONIC SUPPLEMENTARY MATERIAL

The Delphi method is an established procedure to achieve consensus among convenors on a specific topic. It included the following steps:

1. Expert selection

The expert panel consisted of 38 radiologists from 13 countries (Austria, Belgium, Denmark, France, Germany, Greece, India, Italy, Macedonia, New Zealand, Portugal, Spain, and the United Kingdom), with 5 to 35 years of experience in research activity in musculoskeletal interventional procedures and in the scientific evaluation of medical literature. All experts were members of the ESSR Interventions Subcommittee or the ESNR Spine Committee, divided into groups by topic.

2. Literature search, statement drafting, and levels of evidence

The literature search was carried out using the major online medical databases (MEDLINE, Web of Science, EMBASE, and Google), incorporating papers published up to the end of September 2024 by using all relevant search terms to the specific topic assigned to each working group. The experts could also include papers retrieved to evaluate the references of incorporated papers if considered important. Following the literature search, each group defined the evidence-based statements on their topic, applying the Oxford Center of Evidence-Based Medicine criteria of 2011 [1]. The level of evidence may be graded down based on study quality or graded up if there is a large effect size, but, as a general rule, the level of evidence is generally identified as follows [2]:

Level 1: Systematic review of randomized trials

Level 2: Prospective randomized trials

Level 3: Non-randomized controlled cohort or follow-up study

Level 4: Case-series, case–control studies, or historically controlled studies

Level 5: Mechanism-based reasoning, expert opinion

3. Questionnaire preparation and consensus process

The project coordinator revised the drafted statements and disseminated a tool (Google Forms, Google LLC) to all experts by email. All experts accessed the tool to agree or disagree with the drafted statements. Experts were also encouraged to add any comments for each statement about the level of evidence and content. The answers were collected in an electronic spreadsheet (Microsoft Excel, Microsoft) and reviewed by the coordinator, who adjusted the statements per the experts' comments. Afterwards, there was a second round of discussion using the same technique. Any disagreement on the level of evidence and/or statement content remaining after the second round of discussion was resolved via targeted emails sent to the involved experts.

4. Data analysis and paper drafting

Following the rounds of discussion, the statements were circulated again with the experts to attain consensus. The consensus was considered strong when 95% or more of experts agreed with the statement, or it was recognized as broad when more than 80% but less than 95% agreed [3]. The Delphi-based consensus results were used to produce a paper circulated for final approval with all panel members.

References

1. Howick J, Chalmers I, Lind J, et al Oxford Centre for Evidence- Based Medicine 2011 Levels of Evidence. Available at: https://www.cebm.ox.ac.uk/resources/levels-of-evidence/ocebm-levels-of-evidence. Accessed on 5 December 2024.
2. Sconfienza LM, Adriaensen M, Albano D et al (2022) Clinical indications for image-guided interventional procedures in the musculoskeletal system: a Delphi-based consensus paper from the European Society of Musculoskeletal Radiology (ESSR)-part VII, nerves of the lower limb. Eur Radiol 32(3):1456-1464.
3. Săftoiu A, Gilja OH , Sidhu PS (2019) The EFSUMB Guidelines and Recommendations for the Clinical Practice of Elastography in Non-Hepatic Applications: Update 2018. Ultraschall Med 40(4):425-453
